# Evaluation of health impacts of the improved housing conditions on under-five children in the socioeconomically underprivileged families in central India: A 1-year follow-up study protocol

**DOI:** 10.3389/fpubh.2022.973721

**Published:** 2022-09-06

**Authors:** Yogesh Damodar Sabde, Tanwi Trushna, Uday Kumar Mandal, Vikas Yadav, Devojit Kumar Sarma, Satish Bhagwatrao Aher, Surya Singh, Rajnarayan R. Tiwari, Vishal Diwan

**Affiliations:** ^1^Department of Environmental Health and Epidemiology, ICMR-National Institute for Research in Environmental Health, Bhopal, Madhya Pradesh, India; ^2^Department of Molecular Biology, ICMR-National Institute for Research in Environmental Health, Bhopal, Madhya Pradesh, India; ^3^Department of Environmental Monitoring and Exposure Assessment (Air), ICMR-National Institute for Research in Environmental Health, Bhopal, Madhya Pradesh, India; ^4^Department of Environmental Monitoring and Exposure Assessment (Water and Soil), ICMR-National Institute for Research in Environmental Health, Bhopal, Madhya Pradesh, India; ^5^ICMR-National Institute for Research in Environmental Health, Bhopal, Madhya Pradesh, India

**Keywords:** acute morbidities, built environment, housing scheme, India, under-five children

## Abstract

Unacceptable housing conditions prevalent in Indian urban slums adversely affect the health of residents. The Government of India initiated the Basic Services to the Urban Poor (BSUP) as a sub-mission under the Jawaharlal Nehru National Urban Renewal Mission (JNNURM), to provide basic services to the urban poor. As per the available scientific literature, the health effects of such improved housing schemes for the poor have not been studied so far in India, especially in under-five children (0–5 years old) who spend most of their time indoors. The present paper describes the protocol for a follow-up research study proposed to fill this gap. This study, funded by the Indian Council of Medical Research (Sanction No. 5/8-4/9/Env/2020-NCD-II dated 21.09.2021), will be conducted in Bhopal in the central Indian province of Madhya Pradesh for over 2 years. We will recruit 320 under-five children each from Group 1 (Beneficiary families residing in the houses constructed under BSUP) and Group 2 (Slum dwelling families eligible for improved housing but who did not avail of benefit). Eligible children will be recruited in the first household visit. During the same visit, we will record clinical history, examination findings and take anthropometric measurements of participants. We will also collect data regarding socio-economic-environmental parameters of the house. During subsequent monthly follow-up visits, we will collect primary data on morbidity profile, anthropometric details and medical history over 1 year. Approval for the study was obtained from the Institutional Ethics Committee of the National Institute for Research in Environmental Health (No: NIREH/BPL/IEC/2020-21/198, dated 22/06/2020). This study will evaluate the impact of different housing conditions on the health of under-five children. Finding of this research will be beneficial in guiding future housing-related policy decisions in low- and middle-income countries.

## Introduction

Housing conditions affect human health (1). Research from developed countries has shown that better housing conditions can improve health (1–3). Therefore, target 1 of the United Nations' Sustainable Development Goal 11 (SDG 11) emphasized the availability of “safe and affordable housing and basic services for all and upgrade slums by 2030” (4). Target 3 and 5 of SDG 11 are also related to appropriate planning of human settlements to reduce the risk of exposure of the underprivileged population to adverse environmental factors (4). Following these guidelines, many developing countries have initiated public housing programs to meet the housing needs of their poor (5).

In India, a predominantly rural country, research and administrative attention to housing and other basic amenities has traditionally focused on human settlements in underdeveloped rural areas (6). In the meantime, the urban population, including the migrant rural population, has grown exponentially, amounting to almost 31 per cent of the country's population (7, 8). However, urban settlement expansion is often unplanned, with a large proportion of the urban population being either houseless (1.77 million) or dwelling in informal settlements like semi–pucca houses of slums (65–70 million, i.e. 17%) (7).

The improper housing condition prevalent in urban slums in India has a severe adverse effect on the health of its residents. Published studies revealed that it is associated with a higher risk of infections and vector-borne diseases (9). Children under 5 years of age are especially vulnerable since they spend most of their time at home (10). Studies have shown that a higher percentage of slum-dwelling children suffer from acute respiratory infections (ARIs) than non-slum-dwelling children in Indore in Central India (11) and in Gulbarga in South India, where the prevalence of ARIs was 27.25% among under-5 years children (12). Slightly higher ARI prevalence was seen among under-5 years children in urban slums of Nagpur and Vishakhapatnam (32.19 and 36.30%, respectively) (13, 14). Frequent infections contribute to the poor nutritional status of children, have long-term implications on their growth and development (15) and are a leading cause of infant and childhood mortality in developing countries (16).

To tackle the adverse health effects of poor urban housing conditions, the Ministry of Urban Development, Government of India initiated the Basic Services to the Urban Poor (BSUP) sub-mission under the Jawaharlal Nehru National Urban Renewal Mission (JNNURM) in 2005 (8). The primary target of BSUP was the integrated development of urban slums through improved housing at a subsidized price and other related civic amenities (8). Till 2018, under the BSUP submission, 710618 houses have been constructed, 54053 are in progress, and 788953 are sanctioned (17). Through its BSUP, JNNURM has the potential to improve housing conditions and, thus, the health status of the beneficiaries, provided environmental risks of poor housing are controlled while constructing new houses (18). The BSUP model is also likely to be adapted for the projected Housing for All (Urban) mission under Pradhan Mantri Awas Yojana (PMAY) or the Prime Minister Housing Scheme, which is proposed to be implemented from 2015 to 2022 for *in-situ* Rehabilitation of urban slum dwellers (19). Therefore, at this juncture, it is essential to explore the possible health impact of changing housing conditions under BSUP (20). Under-five children (children aged 0–5 years) spend most of their time in and around the home. Their health status can be a sensitive indicator of the built-in environment of their dwellings (10, 21). However, as per the available scientific literature, the effect of subsidized housing schemes on health (especially on children under 5 years of age) has not been studied so far in India. With this background, this study aims to evaluate the impact of different housing conditions on the health of under-five children.

Specifically, it aims to achieve the following objectives:

i. To compare the 1-year incidence of acute morbidities between under-five children of, BSUP beneficiary families and slum-dwelling families of Bhopal.
ii. To explore the association between incidences of acute morbidities and housing conditions while adjusting for other known confounders such as socioeconomic and environmental factors.

## Materials and methods

### Design and duration

**Figure 1:**
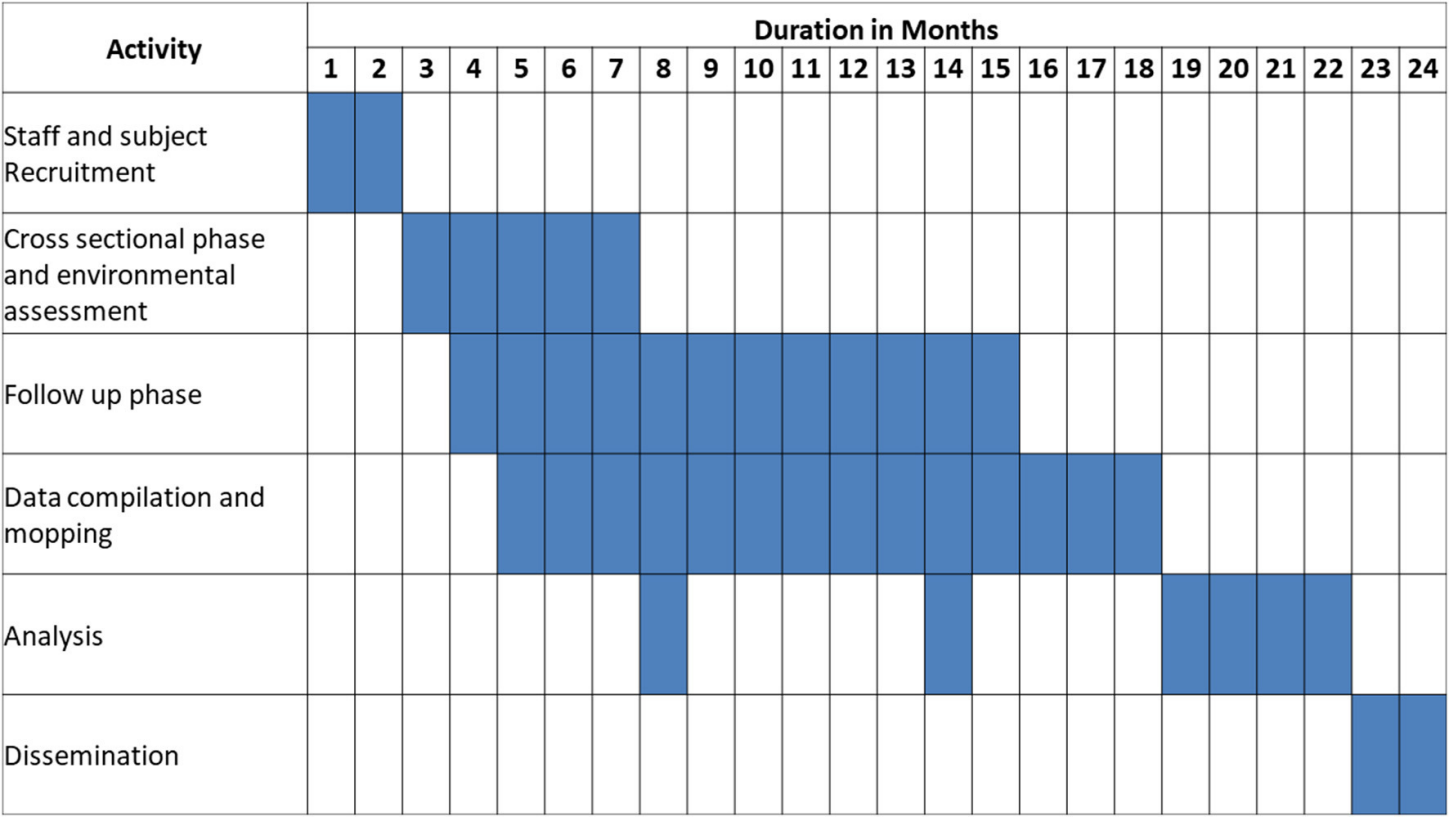
Gantt chart of the project timeline.

This manuscript describes the protocol for a 1-year follow-up study to employ questionnaire-based data collection, biological sampling, and environmental monitoring to ascertain objective achievement. Figure 1 describes schedule of various study methods of this protocol.

### Setting

**Figure 2:**
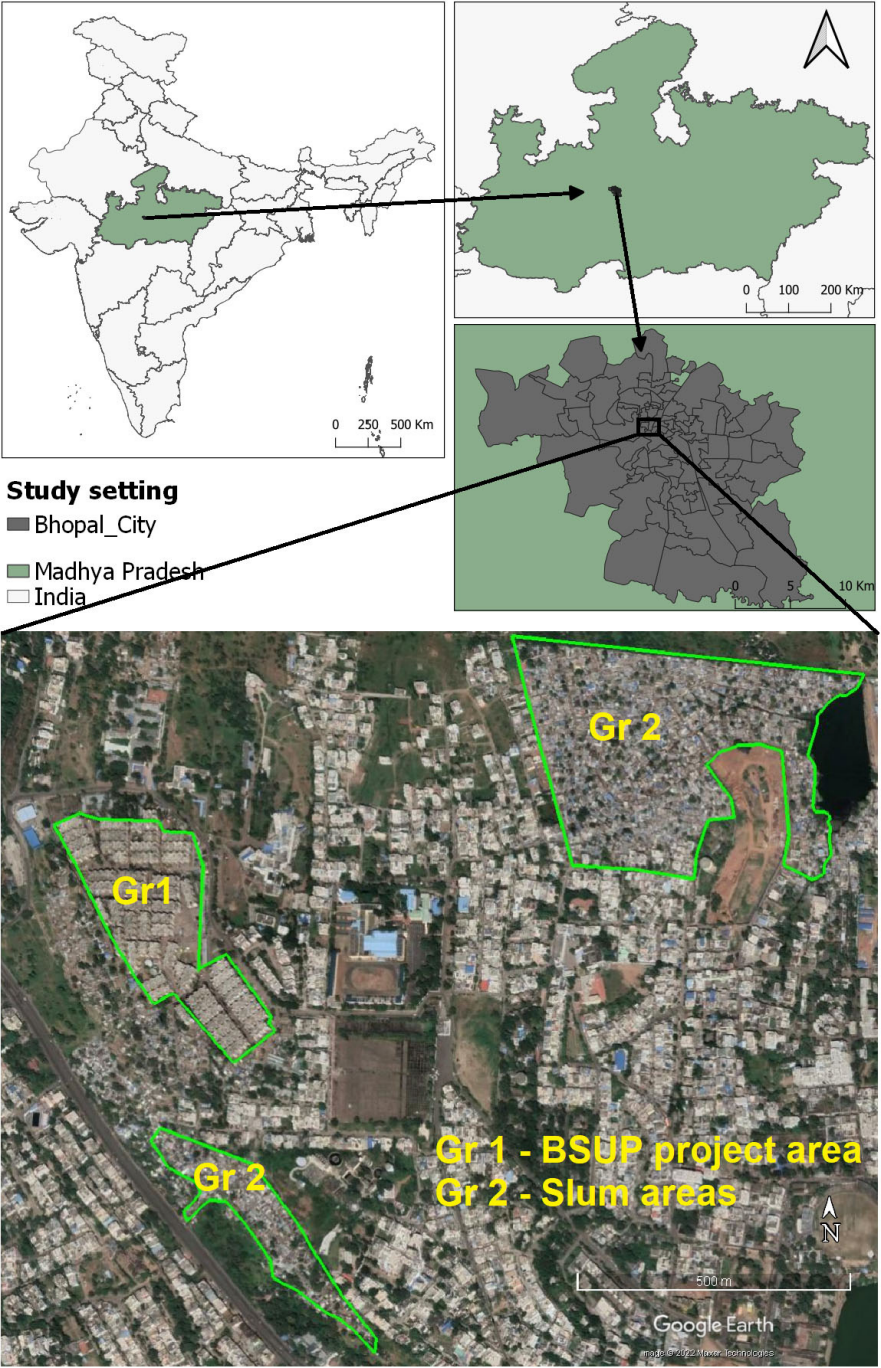
Study area showing the location of Group 1 and Group 2 settings (Note: The image was created by researchers using QGIS Version 3.20, and the open-access image downloaded from Google Earth https://earth.google.com/web/ was used for illustrative purposes).

We will conduct the study in Bhopal city of Bhopal District in the central Indian province of Madhya Pradesh (MP) India (Figure 2). According to the 2011 census of India, MP has a population of almost 72 million (22). The provincial capital, Bhopal city, has a total population of almost 1.8 million (23), of which almost 0.4 million live in slums (24). Under-five children population in Bhopal city is almost 0.2 million (23). Government of India published a status report of BSUP in 2019 titled ‘state wise monitoring report'. The report stated that, 13 projects of BSUP were completed, till January 2019 in the city of Bhopal (25). We have selected the project with the highest number of completed houses (*N* = 3,248) for the current study (Figure 2).

### Study participant groups

Under-five children will be recruited for participation in the current study from the following two groups:

1. Group 1: Beneficiary families residing in the houses constructed under BSUP of JNNURM in the urban areas of Bhopal, MP. The study will be performed in the selected slum resettlement project of BSUP.
2. Group 2: Slum dwelling families of two slum areas located near selected BSUP project mentioned above (Slum dweller families are eligible for the subsidized housing benefits under BSUP of JNNURM scheme. But some dewllers who do not avail/get the scheme continue to dwell in the slum areas).

The following eligibility criteria will be used for recruiting participants:

### Inclusion criteria

1. The family should be a resident of the said household for 1 year.

### Exclusion criteria

1. Known cases of congenital anomalies and moderate or severe “intellectual disability.”
2. Single parenting.
3. Children from families who are migrants, tenants, and paying guests.
4. Family size more than 6.

### Sample size calculation

Previous studies reported that ARI prevalence for under-five children in urban areas ranges from 16.6 to 19.9% (26, 27). In comparison, the ARI prevalence among the under-five children in the slum population ranges from 27.3 to 36.3% (12–14). In this study, we expect that the improved housing conditions offered by BSUP, JNNURM, should reduce the prevalence of ARI from 30 (equivalent to that of slums) to 20% (equal to that of non-slums) according to the available literature. Therefore, to establish a 10% difference in ARI prevalence considering confidence level of 95% (i.e., α error: 5%) and power of 80% (β being 0.2), required sample size was estimated to be 290.5 using following formula:


(1) $$\begin{array}{l} \text{Sample size for the difference in prevalence }(N)\text{ =}\\ \;\frac{{\left(z_{\left(\alpha /2\right)}+z_{\left(\beta \right)}\right)}^{\text{2}}\left({\text{P}}_{1}\left(\text{1 }-\;{\text{P}}_{1}\right)\text{ + }{\text{P}}_{2}\left(\text{1 }-\;{\text{P}}_{2}\right)\right)}{{\left({\text{P}}_{1}\sim {\text{P}}_{2}\;\right)}^{\text{2}}} \end{array}$$

Where P_1_ is the expected population proportion in Group 1 (i.e., 0.2); P_2_ is the expected population proportion in Group 2 (i.e., 0.3); Z_(α/2)_ is the critical value of the normal distribution at α, (i.e., 1.96 for α = 0.05), Z_β_ is the critical value of the normal distribution at β, i.e., 0.842 for β = 0.2. An additional 10% of participants will be enrolled to account for non-response and loss to follow up. Thus, we will enroll 320 children in each group.

### Sampling strategy

We will retrieve the list of children residing in study areas from their respective anganwadis. Anganwadis are community outreach centers under the Integrated Child Development Scheme (ICDS) in India (28), which maintain a list of under-five children for providing nutritional and educational services. We will recruit the eligible children after obtaining written informed parental consent in selected areas of Bhopal city to cover both the participant groups. If two children are selected from the same house, the child selected first will be retained, and the second child will be dropped and replaced by another child with the following random number.

### Data collection

#### Cross-sectional phase (phase 1)

During the initial household visit to recruit eligible children, basic clinical history taking and general physical examination of the children, including anthropometry and detailed household socioeconomic and environmental assessment, will be conducted.

##### Clinical history and examination

The child's anthropometric details (height, weight, skinfold thickness and mid-arm circumference) will be collected following standard guidelines (29). The growth parameters (height, weight, skinfold thickness and mid-arm circumference) will be compared to the WHO growth standards according to age (30). The growth and development of the child will be assessed by estimating age as per achievement of milestones in five different domains, which include gross motor, fine motor, adaptive/cognitive, language and personal—social developmental milestones (31). Immunization status of the child will be recorded and compared with National Immunization Schedule (NIS) under Universal Immunization Programme (UIP) (32). Relevant medical history to assess for the presence of comorbidities and current health ailments, including acute morbidities (respiratory, gastrointestinal and mosquito-borne infections) among under-five children during the last month will be recorded by trained pediatricians. The case definitions laid down by the Integrated Disease Surveillance Project (IDSP) of the Government of India to categorize the morbidities (33) (See Supplementary Annexure 1).

##### Family history

Relevant family history will be collected, such as the number of members in the household and medical illnesses prevalent in the family (for example, history of congenital diseases known to increase the rate of ARI (congenital heart disease) in family members). We will also collect parents' detailed occupational history and tobacco/alcohol consumption (Supplementary Annexure 1).

##### Socioeconomic assessment of household

We will use the 2020 revision of the original Kuppuswamy Scale developed in 1976, a commonly used composite scoring system to assess the household's socioeconomic status (SES) (34). This scale categorizes households into five SES groups, “upper class, upper-middle-class, lower middle class, upper lower and lower socioeconomic class,” based on scores assigned to the household ranging from 3 to 29 according to responses obtained for education and occupation of the head of the household along with total family income per month (34).

##### Environmental assessment

To gather information on the environmental factors that might act as confounding variables in our study, we will conduct a series of assessments listed subsequently (Table 1).

**Table 1:** Proposed sampling frequency of environmental exposure assessment.

**SN**	**Environmental exposure assessment**	**Proposed sampling frequency/number of samples**	**Timeline**
**1**	Household- built environmental assessment	Once	Will be conducted before health assessment starts
**2**	Ambient air quality assessment	For each locality, daily mean values for pollutants over the previous year	Will be retrieved from Central Air pollution control board
**3**	Indoor air quality assessment	64 (32 from each group) households	Will be started after rainy season as per recommended norms
4	Water quality assessment	4 rounds each for 64 (32 from each group) households (Total 256 samples)	First two rounds will be done in pre-monsoon period and next two in post-monsoon period
5	Prevalence of mosquito vector assessment	4 catching stations (2 fixed and 2 random) in each locality for 12 months	Will be conducted throughout the study period

###### Household-built environmental assessment

Details such as the number of rooms, the total area of the house, water supply, sanitation, ventilation, the material used in flooring/walls/roofs, and the fuel used for cooking/heating will be recorded using a predesigned pilot-tested questionnaire prepared for the study. To allow comparison of the study results with national data, relevant questions were adapted from the 'household questionnaire' of the National Family Health Survey, India 2019–2020 (NFHS – 5) (Supplementary Annexure 2).

###### Ambient air pollution assessment

Ambient air pollution data for last year for study area will be retrieved from CBCB (Central Pollution Control Board, India) in the form of daily mean values of particulate matter (PM_10_ and PM_2.5_) and gaseous pollutants (SO_2_ and NO_2_) (35). The obtained data will be compiled and statistically analyzed by comparing it with existing National Ambient Air Quality Standards (NAAQS) (36).

###### Indoor air pollution assessment

To assess indoor air pollution, monitoring of PM_10_, PM_2.5_, PM_1_ and gaseous pollutants will be carried out for 24 h in each representative (10%) household twice a year (once each in pre and post-monsoon seasons). The particulate monitoring will be carried out using a sophisticated dust monitor (Make- TSI Incorporated, USA; Model- DustTrak 8530). Similarly, the indoor air gaseous pollutants, viz., SO_2_ and NO_2_, will be monitored using a multigas monitor (Make- Swan Environmental Pvt. Ltd.; Model: GRI-IAT; Sensor make: Membrapor^®^, Switzerland). The calibration of both monitoring instruments will be ensured before measurement. After successfully monitoring selected households, the recorded data will be retrieved, tabulated and statistically analyzed using the IBM-SPSS-24 statistical package.

###### Water quality assessment

Basic physicochemical parameters of drinking water quality, such as color, odor, taste, pH, electrical conductivity, hardness, turbidity, total dissolved solids (TDS), chloride, fluoride, and nitrates, shall be analyzed at the household level. Bacteriological tests for total and fecal coliform will be performed for a representative number of samples (10%) of different drinking water sources. Sensor-based instruments shall be used for water analysis in the field, and measurements will be conducted four times during the study period. Laboratory experiments shall follow the standard American Public Health Association (APHA) guidelines for determining various water quality parameters (37). Sampling, transportation, and storage of water samples shall also be done per the defined guidelines. Drinking water quality guidelines laid down by the WHO and Bureau of Indian Standards (BIS) will be used to define normal values (38, 39).

###### Prevalence of mosquito vector assessment

Prevalence of potential mosquito vectors of malaria (*Anopheles stephensi* and *Anopheles culicifacies* s.l.) and Dengue/Chikungunya (*Aedes aegypti* and *Aedes albopictus*) will be monitored using standard mosquito collection techniques in four houses (two indexed and two random) in each study groups. *Anopheles* mosquitoes will be collected using CDC light trap installed inside the human dwellings from dusk to dawn. *Aedes* mosquitoes will be collected from inside the houses in the morning and the evening using a mechanical aspirator. All the collected mosquitoes will be identified up to species level using appropriate keys (40, 41). Potential breeding sites for *Anopheles* and *Aedes* mosquitoes will be surveyed in each locality to understand the mosquitogenic potential of the locality. In addition, notified malaria and dengue fever cases will be collected from the district malaria office, Bhopal, for the study period to correlate with vector density and its role in disease transmission.

#### Follow-up phase (phase 2)

The recruited children will be followed-up monthly for 1 year to collect primary data on morbidity profile and growth patterns. In addition, in the past month, anthropometric details and medical history of the child and their family will be recorded in the proforma created for this study (Supplementary Annexure 3).

### Quality control

Necessary precautions will be adopted to maintain the quality of the collected data at a high level. Equipment used in the environmental assessment will be calibrated, and high-quality reagents will be used. Adequate training will be provided to research staff. Questionnaire-based field data collection will be done through standardized tools wherever possible, and author-developed tools will be validated before use.

### Data management and analysis

The principal investigator will be responsible for data management, ensuring that all data generated during this project is stored safely with timely creation and maintenance of backups. Hard copies will be held in a secure archive in the institute, whereas all computer-based data will be kept under password protection to be handled by research staff alone.

Unique identifying code numbers will be assigned to all filled questionnaires, and data from these will be entered into the latest version of Microsoft Excel spreadsheets. During data entry, all morbidities recorded will be coded as per the latest edition of the International Classification of Disease (ICD). In addition, environmental assessment data will be linked to the questionnaire-based socioeconomic, demographic and clinical data. Data entry will be done by junior research team members. It will be supervised by senior researchers who verify at least ten per cent of the entered data for quality assurance.

### Statistical analysis

Our input variables will include duration of stay in improved houses, details of confounders including age, gender, family size, socioeconomic status, family medical history and data of environmental assessment of the households (ambient and indoor air pollution level, type of cooking fuel used, drinking water quality and mosquito vector density). The outcome variables that will be used in the analysis include the incidence rate of acute morbidities (respiratory, gastrointestinal and mosquito-borne) and the growth and development of the child.

The data analysis will be done using SPSS statistics software (Version 26). The analysis will be done using the Intention-to-treat method. If a participant changes his/ her house during the course of follow-up, we will treat the participant as per the original group allotted.

Descriptive statistics such as mean/SD or median/Interquartile range will be presented for continuous variables depending on the data distribution. The normal distribution assumption will be tested using the Kolmogorov Smirnov test. Categorical variables will be summarized using frequency with percentages. The incidence rate will be calculated as the number of ARI episodes divided by the number of children followed over 1 year. Relative risk (RR) for ARI will be calculated as ARI incidence among the BSUP beneficiaries group divided by ARI incidence among the slum dwellers group. Similar calculations will be done for acute gastrointestinal infections and mosquito-borne infections. The RR with 95% CI will also be presented for different factors, such as the gender of the child, socioeconomic status of the family, etc. Finally, univariate and multivariate analyses will compare ARI episodes among the two groups after adjusting for other confounding factors. A multilevel regression modeling analyse is chosen to analyse relative importance of housing characteristics on variation in the incidence of acute morbidities. Individual and housing characteristics that have a significant association with the incidence of acute morbidities in bivariate analysis will be included in multilevel models. The multilevel analysis will conducted at three levels i.e. study groups, housing characteristics and individual characteristics.

### Ethical considerations

Ethics approval has been obtained from the Institutional Ethics Committee (Human), National Institute for Research in Environmental Health (NIREH/BPL/IEC/2020-21/198 date 22nd June 2020). Before the initiation of research activities, written informed consent (Supplementary Annexure 4) for data collection and dissemination through scientific publication will be obtained from the parents or guardians of every study participant. The study findings will be disseminated to benefit the study participants and their families. We will help the children with known cases of congenital anomalies and moderate or severe “intellectual disability” by providing appropriate advice and referral services. However, as these children are more prone to acute infections because of their poor health conditions/underlying comorbidities and this extra burden may mask the effect of housing conditions, data from these children will be excluded from the analysis process. Further, the children found morbid during the survey will be referred to a government hospital located near the study setting.

## Discussion

This protocol describes a prospective study to evaluate the impacts of the different housing conditions on the health of the under-five children while adjusting for the known confounders.

Most research on improved housing conditions' health benefits originates from developed countries. However, an equal number of studies from low and middle-income countries (LMICs) like India is still lacking. Moreover, the presence of environmental risk factors like urban air pollution, solid fuel use for cooking contributing to indoor air pollution, unsafe water and lead exposure is higher in developing countries than in developed ones (42). Thus, a higher burden of infectious disease morbidity is documented among the pediatric population of developing countries (43, 44). Housing benefits estimated by research in high-income countries cannot be extrapolated to LMICs. So the impact of housing conditions on public health needs to be explored in LMICs, especially considering the debate regarding the timing of adoption of housing programs that will prove most beneficial to a developing economy (45). If such research proves that housing improvement can result in a net gain in health outcomes, then additional investment on housing programs like BSUP (India) could be further advocated.

Basic Services to the Urban Poor beneficiaries are expected to have a lower burden of acute morbidities as compared to slum dwellers by virtue of their improved housing conditions. However, few studies have attempted to validate this hypothesis scientifically. Kumar and Singh (46) qualitatively explored the fulfillment of BSUP objectives in Bhopal, India. Similarly, the qualitative impact of slum rehabilitation on household decision-making and energy use from a gender-based perspective has been explored by Sunikka-Blank et al. (47), whereas Kshetrimayum et al. (48), investigated residential satisfaction in association with slum rehabilitation. Furthermore, Gupta and Kavit (49) and Desai et al. (50) analyzed the success and failures of the BSUP implementation in Chandigarh and Nanded, respectively. Quantitative assessment of indoor air quality (51, 52), socio-physical liveability, and built-environment (53) in slums vs. rehabilitated settlements have been conducted in Mumbai, India. Vaid and Evans (54), through their quasi-experimental study, concluded that housing quality could potentially improve women's health and subjective wellbeing. Nevertheless, few of these studies have quantitatively documented the difference in housing environments and its consequent health impacts, especially on vulnerable populations such as children, the elderly, and pregnant women. The current protocol, when implemented, will fill this critical gap in information.

The proposed study will be conducted on under-five children who spend most of their time in their homes and are most susceptible to environmental challenges making them the most sensitive indicator of potential environmental health challenges. The study also engages a comparable control group that will enable investigators to determine whether there are any changes due to the intervention introduced in the form of the housing scheme. The protocol comprehensively explores participants' home environment that will yield baseline information on various environmental factors affecting inhabitants' health. This will be possible through a multidisciplinary research team with expertise ranging from medical disciplines such as public health/epidemiology and pediatrics to environmental sciences, a significant strength of this protocol. Though the BSUP scheme was implemented pan India, the present study protocol will be implemented in a limited geographical area of Bhopal city. However, lessons learnt during this study will aid in conducting similar research in other communities.

## Ethics statement

The studies involving human participants were reviewed and approved by Institutional Ethics Committee (Human), National Institute for Research in Environmental Health, Bhopal, Madhya Pradesh, India (Approval No: NIREH/BPL/IEC/2020-21/198, 22nd June 2020). Written informed consent to participate in this study was provided by the participants' legal guardian/next of kin.

## Author contributions

Conceptualization and funding acquisition: YS. Formal analysis: YS and VY [analysis not yet started]. Methodology and investigation: YS, VD, VY, DS, TT, SA, SS, and UM. Project administration: YS and RT. Visualization and writing—original draft: YS and TT. Writing—review and editing: VD, VY, DS, TT, SA, SS, UM, and RT. All authors met the authorship criteria set forth by the International Committee for Medical Journal Editors and read and approved the final manuscript.

## Funding

This study, on which this protocol is based, was funded by the Indian Council of Medical Research (Extramural funding Sanction letter No. 5/8-4/9/Env/2020-NCD-II dated 21.09.2021). However, the funder had no role in designing the study, decision to publish, or manuscript preparation.

## Conflict of interest

The authors declare that the research was conducted in the absence of any commercial or financial relationships that could be construed as a potential conflict of interest.

## Publisher's note

All claims expressed in this article are solely those of the authors and do not necessarily represent those of their affiliated organizations, or those of the publisher, the editors and the reviewers. Any product that may be evaluated in this article, or claim that may be made by its manufacturer, is not guaranteed or endorsed by the publisher.
