# Supplementary material for: Evaluation of health impacts of the improved housing conditions on under-five children in the socioeconomically underprivileged families in central India: A 1-year follow-up study protocol
Source: Front Public Health. 2022 Sep 6;10:973721. doi: 10.3389/fpubh.2022.973721 (PMC9523261; doi:10.3389/fpubh.2022.973721)
Supplement: Supplementary file 1 [file Data_Sheet_1.pdf]

Supplementary material

Evaluation of health impacts of the improved housing conditions on under five-year children in the socioeconomically underprivileged families in central India: a one-year follow up study protocol

Yogesh Sabde[1*], Tanwi Trushna[1], Uday Mandal[1], Vikas Yadav[1], Devojit Sharma[4], Satish Aher[5], Surya Singh[2], Rajnarayan R Tiwari[6], Vishal Diwan[2,3*].

[1]Department of Environmental Health and Epidemiology, ICMR-National Institute for Research in Environmental Health, Bhopal, Madhya Pradesh, India

[2] Department of Environmental Monitoring And Exposure Assessment (Water and Soil), ICMR-National Institute for Research in Environmental Health, Bhopal, Madhya Pradesh, India

[3]Department of Global Public Health, Karolinska Institutet, Stockholm, Sweden

[4]Department of Molecular Biology, ICMR-National Institute for Research in Environmental Health, Bhopal, Madhya Pradesh, India

[5] Department of Environmental Monitoring And Exposure Assessment (Air), ICMR-National Institute for Research in Environmental Health, Bhopal, Madhya Pradesh, India

[6] ICMR-National Institute for Research in Environmental Health, Bhopal, Madhya Pradesh, India

*Corresponding author (email: vishaldiwan@hotmail.com)

List of annexures

1 Clinical assessment

2 Environmental assessment

3 Follow up

4 Participant Information sheet and consent form

Annexure 1: Clinical assessment

Title: Evaluation of health impacts of the different housing conditions on under five year children in the socioeconomically underprivileged families: a one year follow up study.

Participant Identification No. : _______ Date of interview ______/______/___________
DD M M Y Y YY

Informant (preferably Mother)

a. Name: ____________________________Age __________

b. Education of informant: (completed classes) ___________

c. Contact Number

d. Complete address:
____
____

e. Anganwadi Name and No__________________

f. Type of participant (Locality): Group 1 (BSUP beneficiary) / Group 2 (Urban slum)

Anthropometry

- ☐ Weight: ________Grams
- ☐ Height : ________Cms
- ☐ Mid arm circumference ______Cms
- ☐ Skin fold thickness: ______Cms

Growth and development (age as per Achievement of Milestones)

- ☐ Gross Motor Developmental Milestones ___ months
- ☐ Fine Motor Developmental Milestones ___ months
- ☐ Adaptive/Cognitive Developmental Milestones ___ months
- ☐ Language Developmental Milestones ___ months
- ☐ Personal—Social Developmental Milestones ___ months

Personal history (of Child):

a. Birth History
   i. Place of delivery – Hospital / home

ii. Type of delivery – Vaginal (normal in Hindi) / LSCS
iii. Duration of pregnancy – full term / preterm
iv. History of Hospitalization early neonatal period / post neonatal period / No
v. Number of hospitalization ___
vi. Reason if yes ____
vii. History of previous admission with PEM (Protein energy malnutrition) or in NRC (Nutritional rehabilitation Centre) _____ times (write zero in no history)
viii. Birth weight – if available
ix. 6 month Exclusive Breast Feeding (EBF) – Yes / No
x. If no duration of exclusive breast feeding - ____ months
xi. Age of starting weaning feeds _____

b. Sleep pattern of child
   i. Total duration of sleep on a normal day _____hours
   ii. Does the child sleep during day time on a normal day – Yes / No
   iii. At what time child goes to bed for sleep on a normal day - __ __ PM
c. Play
   i. Does to the child go to play daily – yes / No
   ii. If yes
      1. duration _____ Min
      2. Where does the child goes to play __________

Family history

a. Does any usual resident of your household including you suffer from tuberculosis?
b. Does any usual resident of your household including you suffer from allergic illnesses like Asthma/ urticaria / Any other allergic disorder
   i. Mother ______
   ii. Father ______
   iii. Siblings _____
   iv. Other _____

Medical history

- Number of episode of diarrhea in past one year________
- Number of episodes of respiratory infections in past one year ________
- Number of episodes of any fever (any cause) in past one year ________
- Number of episodes of any fever with rash in past one year ________
- Any history of COVID – 19 till date ________
- Any other relevant medical history ________

Clinical impression

____
____

6. **Immunization** (for age as per NIS under UIP)
    a. Immunization card available for inspection – Yes / No

    b. Vaccination eligibility as per age of child _____
        - ☐ Week 6
        - ☐ Week 10
        - ☐ Week 14
        - ☐ Month 9 – 12
        - ☐ Month 16 - 24

Birth
- ☐ BCG
- ☐ OPV0
- ☐ Hepatitis B – Birth dose

6 week – applicable / NA
- ☐ OPV1
- ☐ Penta1
- ☐ RVV1
- ☐ IPV1
- ☐ PCV1

10 week– applicable / NA
- ☐ OPV2
- ☐ Penta2
- ☐ RVV2

14 week– applicable / NA
- ☐ OPV3
- ☐ Penta3
- ☐ RVV2
- ☐ IPV3
- ☐ PCV2

9-12 month– applicable / NA
- ☐ Measles / MR-1
- ☐ PCV booster
- ☐ Vitamin A1

16-24 month– applicable / NA
- ☐ MR-2
- ☐ DPT – b1
- ☐ OPV – b

Annexure 2 for environmental assessment

Title: Evaluation of health impacts of the different housing conditions on under five year children in the socioeconomically underprivileged families: a one year follow up study.

Date of interview:_____/______/__________
Information to be filled from provided list
Participant identification No. : ________ Name of child_____________
Name mother: __________Name father: if available __________
Locality: Group 1 / Group 2 (Urban slum) Contact No. ______________________
Address ____

8. **House & environmental history**:

8.1. **House:**

8.1.1. No.of rooms: _____
8.1.2. How many rooms in this household are used for sleeping? ___ ___
8.1.3. Floor area(Sq Ft): _______________
8.1.4. Ceiling height in living room ____ (feet)
8.1.5. Porch / balcony – Present / absent
8.1.6. Floor No. – Ground / First floor/ Second floor / Third floor **(dropdown)**Ѳ (Applicable for BSUP only)
8.1.7. Single story / multi-story (Applicable for Slums only)
8.1.8. Main material of the floor (Applicable for Slums only) – Natural / Rudimentary / Finished**(dropdown)** Ѳ

Natural
- ☐ Sand – 12
- ☐ Dung – 13

Rudimentary
- ☐ Raw wood planks - 21
- ☐ Palm/bamboo - 22
- ☐ Brick – 23
- ☐ Stone – 24

Finished
- ☐ Parquet or polished wood - 31
- ☐ Vinyl or asphalt - 32
- ☐ Ceramic tiles - 33
- ☐ Cement - 34
- ☐ Carpet - 35
- ☐ Polished stone/marble/ granite – 36
- ☐ Other – 96

8.1.9. Main material of the roof (Applicable for Slums only) – Natural / Rudimentary / Finished **(dropdown)** Ꙩ

Natural
- No roof – 11
- Thatch/palm leaf/ reed/grass – 12
- Mud – 13
- Sod/mud and grass mixture – 14
- Plastic/polythene sheeting – 15

Rudimentary
- Rustic mat – 21
- Palm/bamboo – 22
- Raw wood planks/timber – 23
- Unburnt brick – 24
- Loosely packed stone – 25

Finished
- Metal/GI - 31
- Wood - 32
- Calamine/cement fiber - 33
- Asbestos sheets - 34
- Rcc/rbc/cement/concrete - 35
- Roofing shingles - 36
- Tiles - 37
- Slate - 38
- Burnt brick - 39
- Other - 96

8.1.10. Main material of the exterior walls (Applicable for Slums only) – Natural / Rudimentary / Finished **(dropdown)** Ꙩ

Natural
- No walls - 11
- Cane/palm/trunks/bamboo - 12
- Mud - 13
- Grass/reeds/thatch - 14

Rudimentary
- Bamboo with mud - 21
- Stone with mud - 22
- Plywood - 23
- Cardboard - 24
- Unburnt brick - 25
- Raw wood/reused wood - 26

Finished
- Cement/concrete - 31
- Stone with lime/cement - 32
- Burnt bricks - 33
- Cement blocks - 34
- Wood planks/shingles - 35
- Gi/metal/asbestos sheets - 36
- Other - 96

8.1.11. Number of Windows
8.1.11.1. Frequency of painting interior walls – twice a year / yearly / less frequently
8.1.11.2. Type of paint material – Chuna / disteper / oil paint
8.1.11.3. Dampness – Present / Absent
8.1.11.4. Moulds – Present / Absent
8.1.12. Does your household own any of the following animals: Cattle (cow, buffalo) / Pigs / Chicken / Goat / dog / cat other pets **______(dropdown)**
8.1.13. Does this household share a sleeping room with (this/these) animal(s)? – Yes / No
8.1.14. Does your household have any mosquito nets that can be used while sleeping? – Yes / No
8.1.15. Did the child sleep under this mosquito net last night? – Yes / No
8.1.16. Practice of burning fire for heat (Alao) in house – Yes / No
8.1.17. Practice of burning of plastic/PVC material in the house – Yes / No
8.1.18. Practice of using mosquito repellent –
8.1.18.1. Type Liquid / coil / mat

8.1.18.2. How many per day _______
8.1.18.3. How many times per day___
8.1.19. Practice of Incense stick
8.1.19.1. How many per day _______
8.1.19.2. How many times per day___

8.2. ***Water**

8.2.1. What is the main source of drinking water for members of your household?

- ☐ Piped into dwelling
- ☐ Piped to yard/plot
- ☐ Piped to neighbour
- ☐ Public tap/standpipe
- ☐ Tube well or borehole
- ☐ Protected dug well
- ☐ Unprotected dug well
- ☐ Protected spring
- ☐ Unprotected spring
- ☐ Rainwater
- ☐ Tanker truck
- ☐ Cart with small tank
- ☐ Surface water (river/dam/Lake/pond/stream/ canal/Irrigation channel)
- ☐ Bottled water
- ☐ Community RO plant
- ☐ Other

8.2.2. What is the main source of water for washing?

- ☐ Piped into dwelling
- ☐ Piped to yard/plot
- ☐ Piped to neighbour
- ☐ Public tap/standpipe
- ☐ Tube well or borehole
- ☐ Protected dug well
- ☐ Unprotected dug well
- ☐ Protected spring
- ☐ Unprotected spring
- ☐ Rainwater
- ☐ Tanker truck
- ☐ Cart with small tank
- ☐ Surface water (river/dam/Lake/pond/stream/ canal/Irrigation channel)
- ☐ Bottled water
- ☐ Community RO plant
- ☐ STP/ recycled
- ☐ Other

8.2.3. Where is the water source located?
8.2.4. How long does it take to go there, get water, and come back in one trip?
8.2.5. In the past two weeks, was the water from this source not available for at least one full day?
8.2.6. Does this household do anything to the water to make it safer to drink?
8.2.7. What does this household usually do to make the water safer to drink? Anything else?

- ☐ Boil
- ☐ Use alum
- ☐ Add bleach/chlorine tablets
- ☐ Strain through a cloth
- ☐ Use water filter (ceramic/sand/cdomposite/etc.)
- ☐ Use electronic purifier
- ☐ Use solar disinfection
- ☐ Let it stand and settle
- ☐ Other

8.2.8. Observe the drinking water storage facility for
- ☐ Lid / cover
- ☐ Tap / dispenser

8.2.9. Observe the hand washing place for
- ☐ presence of water
- ☐ Soap

8.3. ***Sanitation**

8.3.1. What kind of toilet facility do members of your household usually use?

- ☐ Flush or pour flush toilet
- ☐ Flush to piped sewer system
- ☐ Flush to septic tank
- ☐ Flush to pit latrine
- ☐ Flush to somewhere else
- ☐ Flush, don't know where
- ☐ Pit latrine
- ☐ Ventilated improved single pit (vip)/biogas latrine
- ☐ Single pit latrine with slab
- ☐ Single pit latrine without slab/ open pit
- ☐ Twin pit/composting toilet
- ☐ Dry toilet
- ☐ No facility/uses open space or field
- ☐ Other

8.3.2. Where is the toilet facility located?
In own dwelling
In Own yard/plot
Elsewhere

8.3.3. Do you share this toilet facility with other households? Yes / No

8.3.4. Including your own household, how many households use this toilet facility?

8.3.5. What type of drainage facility does your household have?
Closed drainage
Open drainage
Drain in Soak Pit
No drainage

8.3.6. How does this household dispose of the kitchen waste? - Let out into drain / Sewer Open drain Reuse for garden / farming Reuse for other domestic purpose Manual collection

8.3.7. Do you have two dust bins for solid waste segregation?(Need to be observed)

8.4. **Cooking and Food**

8.4.1. Do you have a separate room which is used as a kitchen? Yes / No

8.4.2. Does the room used for cooking have any ventilation (Observation)? Yes / No

8.4.3. In this household, is food cooked on - Stove / a chullah / an open fire

8.4.4. What type of fuel does your household mainly use for cooking? (table frequency in a week)

**Fuel**	Daily	Weekly	Occasionally	Never
Electricity				
LPG, Natural Gas				
Biogas				
Kerosene				
Coal				
Charcoal				
Wood				
Straw Agricultural crop waste				
Dung cake				
No food cooked in house				
Other				

9. Socioeconomic Details:

9.1. Total number of family members: ____________

9.2. Education of the head of the family: _____________ **(dropdown)**

Profession or Honours (7),
Graduate 6
Intermediate or diploma 5
High school certificate 4
Middle school certificate 3
Primary school certificate 2
Illiterate 1

9.3. Occupation of the head of the family: _____________**(dropdown)**

Legislators, Senior Officials & Managers 10
Professionals 9
Technicians and Associate Professionals 8
Clerks 7
Skilled Workers and Shop & Market Sales Workers 6
Skilled Agricultural & Fishery Workers 5
Craft & Related Trade Workers 4
Plant & Machine Operators and Assemblers 3
Elementary Occupation 2
Unemployed 1

9.4. Total family income per month: Rs.___________

9.5. Does your household have a BPL Card? – Yes / No

9.6. Is any usual member of this household covered by a health scheme or health insurance – Yes / No / Do not Know

9.7. If yes, what type of health scheme or health insurance? ____________

9.8. What is the religion of the head of the household? Hindu / Muslim / Christian / Sikh / Buddhist / Jain / No religion / Other

9.9. Category – Scheduled Caste / Scheduled Tribe / OBC / Open

9.10.Tobacco environment
9.10.1. Does anyone smoke inside your house? – Yes / No
9.10.1.1. If yes Specify whom: Father / Mother / any other (Multiple response)
9.10.1.2. How often does anyone (Smoker) smoke inside your house? Daily / weekly / monthly / less than monthly
9.10.1.3. Products used in which form: bidis / cigarettes any other (specify) _________
9.10.1.4. Does/ did smokers used to smoke in presence of child? Yes / No
9.10.1.4.1. If yes, How many bidis / cigarettes per day? (Cumulative of all smokers in family)___

9.11.Alcohol Environment:
9.11.1. Does any member of this household consume alcohol – Yes / No
9.11.1.1. If yes; specify whom: Father / Mother / any other
9.11.1.2. Frequency of alcohol consumption – Daily / weekly / monthly / less than monthly

10. **Family history**
10.1.*Does any usual resident of your household including you suffer from tuberculosis?
10.2. Does any usual resident of your household including you suffer from allergic illnesses like Asthma/ urticaria / Any other allergic disorder
10.2.1. Mother ______
10.2.2. Father ______
10.2.3. Siblings _____
10.2.4. Other _____

11. Occupational History of parents
11.1.What is main current occupation of Mother? (Single response)
1. Currently unemployed since _______ months.
2. Agricultural laborer
3. Non-agricultural laborer
4. Domestic servant
5. Skilled worker
6. Unskilled worker
7. Petty business/large business/small shop/self employed
8. Service (Government/Private)
9. Student
10. Truck driver / helper
11. Local transport worker (auto/taxi/driver, hand cart puller, rickshaw puller)
12. Hotel staff
13. Agricultural cultivator/landholder
14. Retired
15. Housewife

11.1.1. If response is 2 to 13
a. Age of joining present occupation (yrs):
b. Total length of service (yrs):
c. Working hours : Per day

d. Weekly / periodic off
e. Whether child accompanies mother to the work place – Yes / No

3.e.1. How frequently _____

11.2. Father

11.2.1. What is main current occupation of Father?

1. Currently unemployed since _______ months.
2. Agricultural laborer
3. Non-agricultural laborer
4. Domestic servant
5. Skilled worker
6. Unskilled worker
7. Petty business/large business/small shop/self employed
8. Service (Government/Private)
9. Student
10. Truck driver / helper
11. Local transport worker (auto/taxi/driver, hand cart puller, rickshaw puller)
12. Hotel staff
13. Agricultural cultivator/landholder
14. Retired

11.2.2. If response is 2 to 13

a. Age of joining present occupation (yrs):
b. Total length of service (yrs):
c. Working hours: Per day:

Observation check list

11.2.3. Flies in house – Yes /No

11.2.4. Have you observed practice of dumping of solid waste material nearby the house – Yes / No

11.2.5. Have you observed practice of burning of solid waste material nearby the house – Yes / No

Annexure 3 for collecting followup data

Title: Evaluation of health impacts of the different housing conditions on under five year children in the socioeconomically underprivileged families: a one year follow up study.

Participant identification No. : ________ Date of interview:______/______/__________
Name of child______________ Name mother: ___________Name father: if available ___________
Locality: Group 1 / Group 2 (Urban slum) Contact No. _______________________
Address ____

Details of illness reported during past one month (since last visit) (including presenting complaints if any):

Diagnosis*	ICD code	No of episodes	Duration of illness in episode no.				
			Episode 1	Episode 2	Episode 3	Episode 4	Episode 5
ARI							
Pneumonia							
ADD							
Dysentry							
Malaria							
Dengue							
Chikungunya							
COVID -19 (RTPCR / RAT +Ve)							
Other							

* Modified Case Definitions of the P form under **Integrated Disease Surveillance Project (**IDSP) (July 2016)

History of similar illness among siblings or friends – Yes / No

____

Other relevant details if any

____

____

____

____

Annexure 4: Participant Information Sheet and Informed consent form

Evaluation of health impacts of the improved housing conditions on under five year children in the socioeconomically underprivileged families: a one year follow up study.

ICMR- National Institute for Research in Environmental Health (ICMR-NIREH), Bhopal

Participant Information Sheet – (English)

Version 1.0 Date 10.11.2019

Warm Greetings!

We invite you and your child to take part in a research study being conducted by Indian Council of Medical Research - National Institute for Research in Environmental Health (ICMR-NIREH), Bhopal. The study details as well as your rights as a participant, are described below;

1. Rationale (Study Purpose)

In order to cope with problems emerging as a result of rapid urban growth, Jawaharlal Nehru National Urban Renewal Mission (JNNURM) was launched by Government of India, under Ministry of Urban Development. JNNURM has an important Sub-Mission for Basic Services to the Urban Poor (BSUP) which has potential to improve housing conditions and thus, health status of the beneficiaries, provided environmental risks of poor housing are controlled while constructing new houses. Therefore it is essential to explore the possible health impact of changing housing conditions under JNNURM - BSUP. The present study will compare the burden of acute morbidities among the Under five children (0 to 5 years) from two study groups while adjusting for the known confounders. Group 1 will include Under five children (0 to 5 years) belonging to beneficiary families residing in the houses constructed under BSUP of JNNURM in the urban areas of Bhopal, MP. Second Group will include Under five children (0 to 5 years) belonging to slum dwelling families of Bhopal, MP which were otherwise eligible for the subsidized housing benefits under BSUP of JNNURM scheme but actually not benefitted. Environmental assessment, will be done in representative sample of households/locality from each group on quarterly interval.

2. Procedures

We are approaching you because; you are a beneficiary / potential beneficiary of JNNURM-BSUP scheme. If you agree to participate in this surveillance, then we will ask you few questions about your personal and family background, household details, exposure and medical history. We will follow your child for a period of one year to record occurrence of any incident acute illness. If you allow we will take 2-3 ml of blood sample of the child during sickness episode. Samples will be

drawn taking all precautions to cause minimum discomfort to you. Blood samples will then be tested for Immune Profile of the child.

3. **Risks of participation**

You may experience some discomforts while participating the interviews. There might be mild discomfort during the collection of blood at the site of blood collection. We will employ trained personnel to minimize the discomfort.

4. **Benefits**

Your participation will help us to understand the health impacts of the improved housing conditions on under five year children in the socioeconomically underprivileged families. After analyzing the results, a report will be shared with the State /District level health officials so that adequate preventive measures can be ensured in future. You need not have to pay for the tests and any further management will be offered by the State health authorities as per the State guidelines.

5. **Participant rights**

Taking part in this surveillance is voluntary. You can choose not to take part. You can choose not to answer a specific question. You can also stop answering these questions at any time without having to provide a reason. This will not affect any of your rights including right to have benefits of any Government scheme for which you are otherwise eligible.

6. **Privacy and confidentiality**

Appropriate care will be taken to safeguard your identity and personal information. Your data will be collected using a structured interview schedule by a trained interviewer. Your specimens will be processed using code numbers. The left-over samples will be preserved for future research. All the information provided by you will be kept confidential and stored securely. Data related to you will be used only for scientific purposes.

7. **Compensation**

No compensation in cash or kind will be paid to you for taking part in this surveillance.

8. **Contact details**

If you wish to find out more about this survey, you can ask me all the questions you want and seek clarifications. If you have any questions or concerns, you can contact the Principal Investigator **Dr. Yogesh Sabde**, Scientist E, ICMR-NIREH, Bhopal on**, Phone No.** 9479787480 **Email: yogesh.sabde.nireh@gov.in**

If you are willing to participate, we will go ahead now.

Evaluation of health impacts of the improved housing conditions on under five year children in the socioeconomically underprivileged families: a one year follow up study.

ICMR- National Institute for Research in Environmental Health (ICMR-NIREH), Bhopal

Informed consent form – (English)

Version 1.0 **Date 10.11.2019**

Participant type: **Group 1 :** ☐ **Group 2 :** ☐

Participant ID no:

Name of Child: ____Age ___________

Name of Name of the parent / legal guardian: ____________________________ Age___________

I have read the participant information sheet (It has been read to me), and the details provided in the participant information sheet and consent form have been explained to me. I was given the opportunity to ask questions and they have been answered to my satisfaction. I was given the time and freedom to decide to participate at my own free will.

I hereby consent voluntarily to let my child take part in this research project and understand that I have the right to refuse to answer any question and withdraw from the survey at any time without giving any reason. I am also aware that my withdrawal from the study will not affect my future medical care. By signing the consent, I agree to the use of the data provided by me for surveillance/research purposes and consent to be contacted for further information

Kindly tick (✔) the relevant option for consenting

( ) I consent for the survey team to interview and contact me for follow-up related to the survey

( ) I consent for providing the blood sample of my child during sickness

Date	Name of the parent / legal guardian	Signature/thumb impression of the parent / a legal guardian

[The literate witness selected by the participant must sign the informed consent form. The witness should not have any relationship with the research team; If the participant doesn't want to disclose his / her participation details to others, in view of respecting the wishes of the participant, he / she can be allowed to waive from the witness procedure (This is applicable to literate participant ONLY). This should be documented by the study staff by getting signature from the prospective participant]

____

"I have witnessed the accurate reading of the consent form to the potential participant and the individual has had opportunity to ask questions. I confirm that the individual has given consent freely"

Date	Name of the witness	Signature of the witness
Date	Name of the investigator/ person obtaining consent	Signature of the investigator/person obtaining consent
